# BO-LSTM: classifying relations via long short-term memory networks along biomedical ontologies

**DOI:** 10.1186/s12859-018-2584-5

**Published:** 2019-01-07

**Authors:** Andre Lamurias, Diana Sousa, Luka A. Clarke, Francisco M. Couto

**Affiliations:** 1 LASIGE, Faculdade de Ciências, Universidade de Lisboa, Lisboa, 1749 016 Portugal; 2 University of Lisboa, Faculty of Sciences, BioISI - Biosystems & Integrative Sciences Institute, Campo Grande, C8 bdg, Lisboa, 1749 016 Portugal

**Keywords:** Text mining, Drug-drug interactions, Deep learning, Long short term memory, Relation extraction

## Abstract

**Background:**

Recent studies have proposed deep learning techniques, namely recurrent neural networks, to improve biomedical text mining tasks. However, these techniques rarely take advantage of existing domain-specific resources, such as ontologies. In Life and Health Sciences there is a vast and valuable set of such resources publicly available, which are continuously being updated. Biomedical ontologies are nowadays a mainstream approach to formalize existing knowledge about entities, such as genes, chemicals, phenotypes, and disorders. These resources contain supplementary information that may not be yet encoded in training data, particularly in domains with limited labeled data.

**Results:**

We propose a new model to detect and classify relations in text, BO-LSTM, that takes advantage of domain-specific ontologies, by representing each entity as the sequence of its ancestors in the ontology. We implemented BO-LSTM as a recurrent neural network with long short-term memory units and using open biomedical ontologies, specifically Chemical Entities of Biological Interest (ChEBI), Human Phenotype, and Gene Ontology. We assessed the performance of BO-LSTM with drug-drug interactions mentioned in a publicly available corpus from an international challenge, composed of 792 drug descriptions and 233 scientific abstracts. By using the domain-specific ontology in addition to word embeddings and WordNet, BO-LSTM improved the F1-score of both the detection and classification of drug-drug interactions, particularly in a document set with a limited number of annotations. We adapted an existing DDI extraction model with our ontology-based method, obtaining a higher F1 score than the original model. Furthermore, we developed and made available a corpus of 228 abstracts annotated with relations between genes and phenotypes, and demonstrated how BO-LSTM can be applied to other types of relations.

**Conclusions:**

Our findings demonstrate that besides the high performance of current deep learning techniques, domain-specific ontologies can still be useful to mitigate the lack of labeled data.

## Background

Current relation extraction methods employ machine learning algorithms, often using kernel functions in conjunction with Support Vector Machines [1, 2] or based on features extracted from the text [3]. In recent years, deep learning techniques have obtained promising results in various Natural Language Processing (NLP) tasks [4], including relation extraction [5]. These techniques have the advantage of being easily adaptable to multiple domains, using models pre-trained on unlabeled documents [6]. The success of deep learning for text mining is in part due to the high quantity of raw data available and the development of word vector models such as word2vec [7] and GloVe [8]. These models can use unlabeled data to predict the most probable word according to the context words (or vice-versa), leading to meaningful vector representations of the words in a corpus, known as word embeddings.

A high volume of biomedical information relevant to the detection of Adverse Drug Reactions (ADRs), such as Drug-Drug Interactions (DDI), is mainly available in articles and patents [9]. A recent review of studies about the causes of hospitalization in adult patients has found that ADRs were the most common cause, accounting for 7% of hospitalizations [10]. Another systematic review focused on the European population, identified that 3.5% of hospital admissions were due to ADRs, while 10.1% of the patients experienced ADRs during hospitalization [11].

**Fig. 1:**
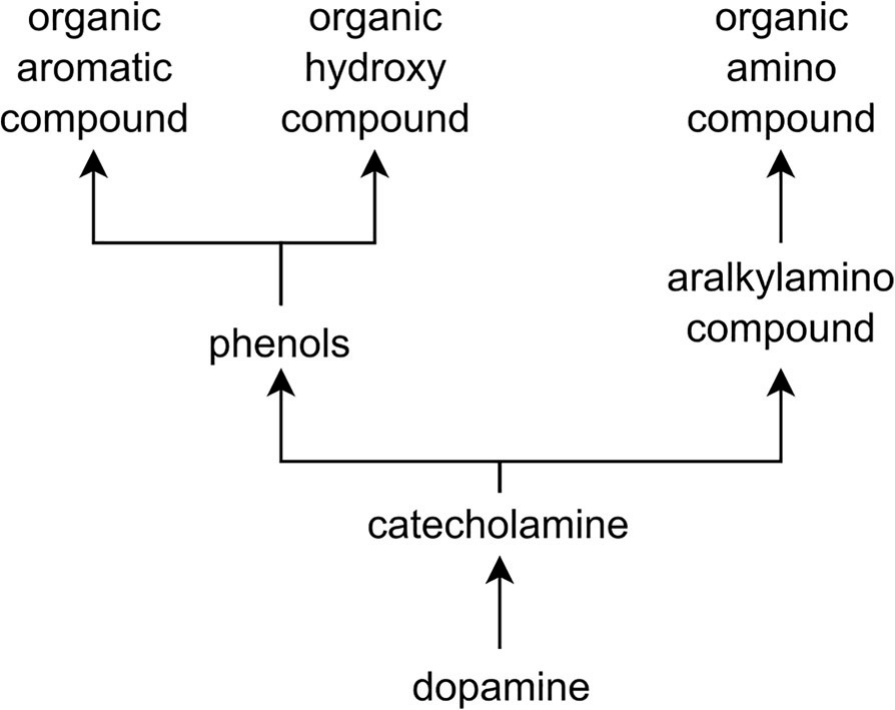
An excerpt of the ChEBI ontology showing the first ancestors of dopamine, using “is-a” relationships

The knowledge encoded in the ChEBI (Chemical Entities of Biological Interest) ontology is highly valuable for detection and classification of DDIs, since it provides not only the important characteristics of each individual compound but also, more importantly, the underlying semantics of the relations between compounds. For instance, dopamine (CHEBI:18243), a chemical compound with several important roles in the brain and body, can be characterized as being a catecholamine (CHEBI:33567), an aralkylamino compound (CHEBI:64365) and an organic aromatic compound (CHEBI:33659) (Fig. 1). When predicting if a certain drug interacts with dopamine, its ancestors will provide additional information that is not usually directly expressed in the text. While the reader can consult additional materials to better understand a biomedical document, current relation extraction models are trained solely on features extracted from the training corpus. Thus, ontologies confer an advantage to relation extraction models due to the semantics encoded in them regarding a particular domain. Since ontologies are described in a common machine-readable format, methods based on ontologies can be applied to different domains and incorporated with other sources of knowledge, bridging the semantic gap between relation extraction models, data sources, and results [12].

### Deep learning for biomedical NLP

Current state-of-the-art text mining methods employ deep learning techniques, such as Recurrent Neural Networks (RNN), to train classification models based on word embeddings and other features. These methods use architectures composed of multiple layers, where each layer attempts to learn a different kind of representation of the input data. This way, different types of tasks can be trained using the same input data. Furthermore, there is no need to manually craft features for a specific task.

Long Short-Term Memory (LSTM) networks have been proposed as an alternative to regular RNN [13]. LSTMs are a type of RNN that can handle long dependencies, and thus are suitable for NLP tasks, which involve long sequences of words. When training the weights of an RNN, the contribution of the gradients may vanish while propagating for long sequences of words. LSTM units account for this vanishing gradient problem through a gated architecture, which makes it easier for the model to capture long-term dependencies. Recently, LSTMs have been applied to relation extraction tasks in various domains. Miwa and Bansal [14] presented a model that extracted entities and relations based on bidirectional tree-structured and sequential LSTM-RNNs. The authors evaluated this model on three datasets, including the SemEval 2010 Task 8 dataset, which defines 10 general semantic relations types between nominals [15].

Bidirectional LSTMs have been proposed for relation extraction, obtaining better results than one-directional LSTMs on the SemEval 2010 dataset [16]. In this case, at each time step, there are two LSTM layers, one that reads the sentence from left to right, and another that reads from right to left. The output of both layers is combined to produce a final score.

The model proposed by Xu et al. [17] combines Shortest Dependency Paths (SDP) between two entities in a sentence with linguistic information. SDPs are informative features for relations extraction since these contain the words of the sentence that refer directly to both entities. This model has a multichannel architecture, where each channel makes use of information from a different source along the SDP. The main channel, which contributes the most to the performance of the model, uses word embeddings trained on the English Wikipedia with word2vec. Additionally, the authors study the effect of adding channels consisting of the part-of-speech tags of each word, the grammatical relations between the words of the SDP, and the WordNet hypernyms of each word. Using all four channels, the F1-score of the SemEval 2010 Task 8 was 0.0135 higher than when using only the word embeddings channel. Although WordNet can be considered an ontology, its semantic properties were not integrated in this work, since only the word class is extracted, and the relations between classes are not considered.

Deep learning approaches to DDI classification have been proposed in recent years, using the SemEval 2013: Task 9 DDI extraction corpus to train and evaluate their performance. Zhao et al. [18] proposed a syntax convolutional neural network for DDI extraction, using word embeddings. Due to its success on other domains, LSTMs have also been used for DDI extraction [19–22]. Xu et al. [21] proposed a method that combines domain-specific biomedical resources to train embedding vectors for biomedical concepts. However, their approach uses only contextual information from patient records and journal abstracts and does not take into account the relations between concepts that an ontology provides. While these works are similar to ours, we present the first model that makes use of a domain-ontology to classify DDIs.

### Ontologies for biomedical text mining

While machine learning classifiers trained on word embeddings can learn to detect relations between entities, these classifiers may miss the underlying semantics of the entities according to their respective domain. However, the semantics of a given domain are, in some cases, available in the form of an ontology. Ontologies aim at providing a structured representation of the semantics of the concepts in a domain and their relations [23]. In this paper, we consider a domain-specific ontology as a directed acyclic graph where each node is a concept (or entity) of the domain and the edges represent known relations between these concepts [24]. This is a common representation of existing biomedical ontologies, which are nowadays a mainstream approach to formalize knowledge about entities, such as genes, chemicals, phenotypes, and disorders.

Biomedical ontologies are usually publicly available and cover a large variety of topics related to Life and Health Sciences. In this paper, we use ChEBI, an ontology for chemical compounds with biological interest, where each node corresponds to a chemical compound [25]. The latest release of ChEBI contains nearly 54k compounds and 163k relationships. Note that, the success of exploring a given biomedical ontology for performing a specific task can be easily extended to other topics due to the common structure of biomedical ontologies. For example, the same measures of metadata quality have been successfully applied to resources annotated with different biomedical ontologies [26].

Other authors have previously combined ontological information with neural networks, to improve the learning capabilities of a model. Li et al. [27] mapped each word to a WordNet sense disambiguation to account for the different meanings that a word may have and the relations between word senses. Ma et al. [28] proposed the LSTM-OLSI model, which indexes documents based on the word-level contextual information from the DBpedia ontology and document-level topic modeling. Some authors have explored graph embedding techniques, converting relations to a low dimensional space which represents the structure and properties of the graph [29]. For example, Kong et al. [30] combined heterogeneous sources of information, such as ontologies, to perform multi-label classification, while Dasigi et al. [31] presented an embedding model based on ontology concepts to represent word tokens.

However, few authors have explored biomedical ontologies for relation extraction. Textpresso is a project that aims at helping database curation by automatically extracting biomedical relations from research articles [32]. Their approach incorporates an internal ontology to identify which terms may participate in relations according to their semantics. Other approaches measure the similarity between the entities and use the value as a feature for a machine learning classifier [33]. One of the teams that participated in the BioCreative VI ChemProt task used ChEBI and Protein Ontology to extract additional features for a neural network model that extracted relation between chemicals and proteins [34]. To the best of our knowledge, our work is the first attempt at incorporating ancestry information from biomedical ontologies with deep learning to extract relations from text.

In this manuscript, we propose a new model, BO-LSTM that can explore domain information from ontologies to improve the task of biomedical relation extraction using deep learning techniques. We compare the effect of using ChEBI, a domain-specific ontology, and WordNet, a generic English language ontology, as external sources of information to train a classification model based on LSTM networks. This model was evaluated on a publicly available corpus of 792 drug descriptions and 233 scientific abstracts annotated with DDIs relevant to the study of adverse drug effects. Using the domain-specific ontology in addition to word embeddings and WordNet, BO-LSTM improved the F1-score of the classification of DDIs by 0.0207. Our model was particularly efficient with document types that were less represented in the training data. Moreover, we improved the F1-score of an existing DDI extraction model by 0.022 by adding our proposed ontology information, and demonstrated its applicability to other domains by generating a corpus of gene-phenotype relations and training our model on that corpus. The code and results obtained with the model can be found on our GitHub repository (https://github.com/lasigeBioTM/BOLSTM), while a Docker image is also available (https://hub.docker.com/r/andrelamurias/bolstm), simplifying the process of training new classifiers and applying them to new data. We also made available the corpus produced for gene-phenotype relations, where each entity is mapped to an ontology concept. These results support our hypothesis that domain-specific information is useful to complement data-intensive approaches such as deep learning.

## Methods

**Fig. 2:**
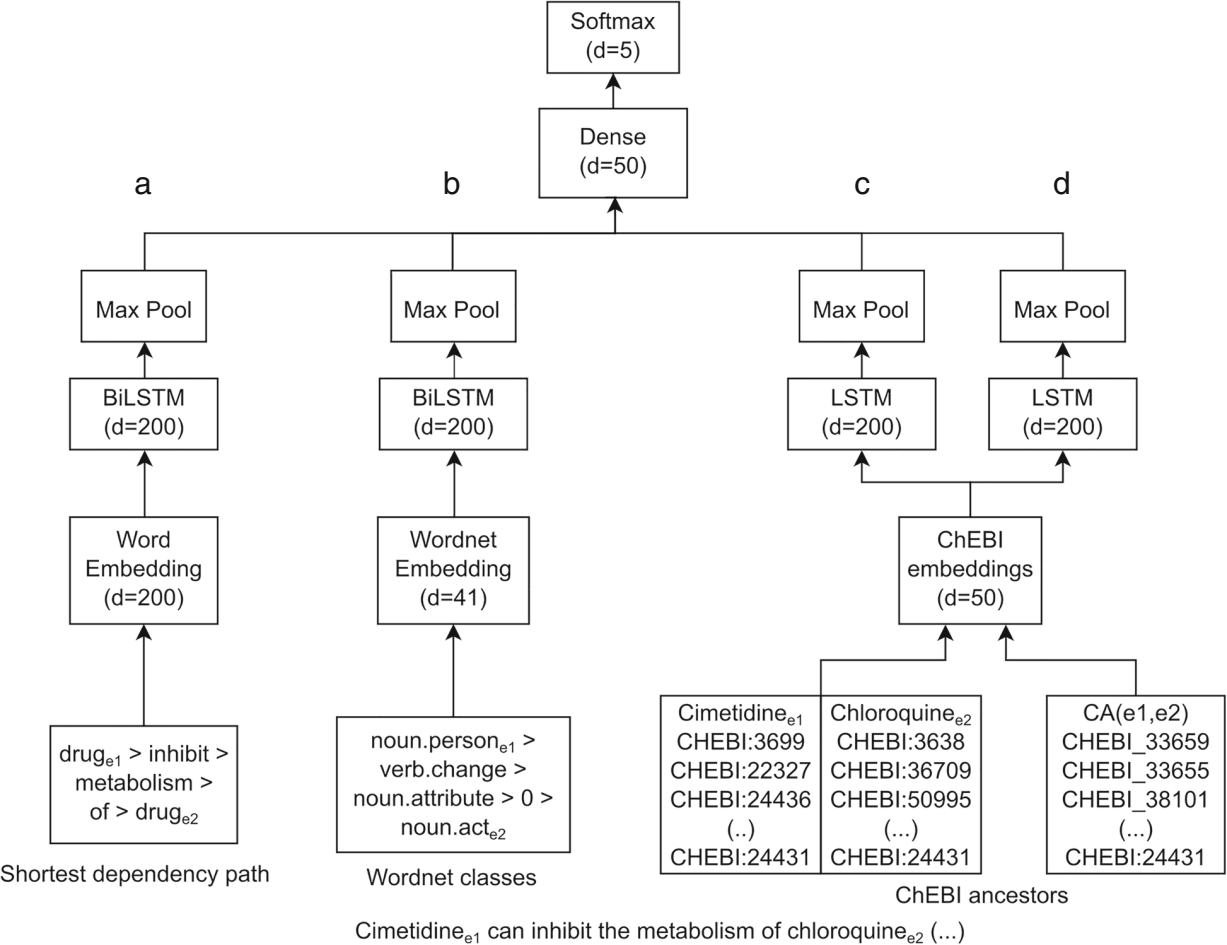
BO-LSTM Model architecture, using a sentence from the Drug-Drug Interactions corpus as an example. Each box represents a layer, with an output dimension, and merging lines represent concatenation. We refer to **a** as the Word embeddings channel, **b** the WordNet channel and **c** the ancestors concatenation channel and **d** the common ancestors channel

In this section, we describe the proposed BO-lSTM model in detail, as shown in Fig. 2, with a focus on the aspects that refer to the use of biomedical ontologies.

### Data preparation

The objective of our work is to identify and classify relations between biomedical entities found in natural language text. We assume that the relevant entities are already recognized. Therefore, we process the input data in order to generate instances to be classified by the model. Considering the set of entities *E* mentioned in a sentence, we generate $\binom {E}{2}$ instances of that sentence. We refer to each instance as a candidate pair, identified by the two entities that constitute that pair, regardless of the order. A relation extraction model will assign a class to each candidate pair. In some cases, it is enough to simply classify the candidate pairs as negative or positive, while in other cases different types of positive relations are considered.

An instance should contain the information necessary to classify a candidate pair. Therefore, after tokenizing each sentence, we obtain the Shortest Dependency Path (SDP) between the entities of the pair. For example, in the sentence “Laboratory Tests Response to Plenaxis _*e*1_ should be monitored by measuring serum total testosterone _*e*1_ concentrations just prior to administration on Day 29 and every 8 weeks thereafter”, the shortest path between the entities would be Plenaxis - Response - monitored - by - measuring - concentrations - testosterone. For both tokenization and dependency parsing, we use the spaCy software library (https://spacy.io/). The text of each entity that appears in the SDP, including the candidate entities, is replaced by the generic string to reduce the effect of specific entity names on the model. For each element of the SDP, we obtain the WordNet hypernym class using the tool developed by Ciaramita and Altun [35].

To focus our attention on the effect of the ontology information, we use pre-trained word embedding vectors. Pyysalo et al. [36] released a set of vectors trained on PubMed abstracts (nearly 23 million) and PubMed Central full documents (nearly 700k), with the word2vec algorithm [7]. Since these vectors were trained on a large biomedical corpus, it is likely that its vocabulary will contain more words relevant to the biomedical domain than the vocabulary of a generic corpus.

We match each entity to an ontology concept so that we can then obtain its ancestors. Ontology concepts contain an ID, a preferred label, and, in most cases, synonyms. While pre-processing the data, we match each entity to the ontology using fuzzy matching. The adopted implementation uses the Levenshtein distance to assign a score to each match.

Our pipeline first attempts to match the entity string to a concept label. If the match has a score equal to or higher than 0.7 (determined empirically), we accept that match and assign the concept ID to that entity. Otherwise, we match to a list of synonyms of ontology concepts. If that match has a score higher than the original score, we assign the ID of the matched synonym to the entity, otherwise, we revert to the original match. It is preferable to match to a concept label since these are more specific and should reflect the most common nomenclature of the concepts. This way, every entity was matched to a ChEBI concept, either to its preferred label or to a synonym. Due to the automatic linking method used, we cannot assume that every match is correct, but fuzzy matching has been used for similar purposes [37], so we can assume that the best match is chosen. We matched 9020 unique entities to the preferred label and 877 to synonyms, and 1283 unique entities had an exact match to either a preferred label or synonym.

The DDI corpus used to evaluate our method has a high imbalance of positive and negative relations, which hinders the training of a classification model. Even though only entities mentioned in the same sentence are considered as candidate DDIs, there is still a ratio of 1:5.9 positive to negative instances. Other authors have suggested reducing the number of negative relations through simple rules [38, 39]. We excluded from training and automatically classify as negative the pairs that fit the following rules:

- entities have the same text (regardless of case): in nearly every case a drug does not interact with itself;
- the only text between the candidate pair is punctuation: consecutive entities, in the form of lists and enumerations, are not interacting, as well as instances where the abbreviation of an entity is introduced;
- both entities have anti-positive governors: we follow the methodology proposed by [38], where the headwords of entities that do not interact are used to filter less informative instances.

With this filtering strategy, we used only 15,697 of the 27,792 pairs of the training corpus, obtaining a ratio of 1:3.5 positive to negative instances.

We developed a corpus of 228 abstracts annotated with human phenotype-gene relations, which we refer to as the HP corpus, to demonstrate how our model could be applied to other relation extraction tasks. This corpus was based on an existing corpus that were manually annotated with 2773 concepts of the Human Phenotype Ontology [40], corresponding to 2170 unique concepts. The developers of the Human Phenotype Ontology made available a file that links phenotypes and genes that are associated with the same diseases. Each gene of this file was automatically annotated on the HP corpus through exact string matching, resulting in 360 gene entity mentions. Then, we assumed that every gene-phenotype pair that co-occurred in the same sentence was a positive instance if this relation existed in the file. While the phenotype entities were manually mapped to the Human Phenotype Ontology, we had to employ an automatic method to obtain the most representative Gene Ontology [41, 42] concept of each gene, giving preference to concepts inferred from experiments. We applied the same pre-processing steps as for the DDI corpus, except for entity matching and negative instance filtering. This corpus is available at https://github.com/lasigeBioTM/BOLSTM/tree/master/HP%20corpus.

### BO-LSTM model

The main contribution of this work is the integration of ontology information with a neural network classification model. A domain-specific ontology is a formal definition of the concepts related to a specific subject. We can define an ontology as a tuple <*C*,*R*>, where C is the set of concepts and R the set of relations between the concepts, where each relation is a pair of concepts (*c*_1_,*c*_2_) with *c*_1_,*c*_2_∈*E*. In our case, we consider only subsumption relations (is-a), which are transitive, i.e. if (*c*_1_,*c*_2_)∈*R* and (*c*_2_,*c*_3_)∈*R*, then we can assume that (*c*_1_,*c*_3_) is a valid relation. Then, the ancestors of concept *c* are given by

1 $$ Anc(c) = {a : (c, a) \in T}  $$

where *T* is the transitive closure of *R* on the set *E*, i.e., the smallest relation set on *E* that contains *R* and is transitive. Using this definition, we can define the common ancestors of concepts *c*_1_ and *c*_2_ as

2 $$ CA\left(c_{1}, c_{2}\right) = Anc\left(c_{1}\right) \cap Anc \left(c_{2}\right)  $$

and the concatenation of the ancestors of concepts *c*_1_ and *c*_2_ as

3 $$ Conc\left(c_{1}, c_{2}\right) = Anc \left(c_{1}\right) \oplus Anc\left(c_{2}\right)  $$

We consider two types of representations of a candidate pair based on the ancestry of its elements: the first consisting of the concatenation of the sequence of ancestors of each entity; and second, consisting of the common ancestors between both entities. Each set of ancestors is sorted by its position in the ontology so that more general concepts are in the first positions and the final position is the concept itself. Common ancestors are also used in some semantic similarity measures [43–45], since they normally represent the common information between two concepts. Due to the fact that in some cases there can be almost no overlap between the ancestors of two concepts, the concatenation provides an alternative representation.

We first represent each ontology concept as a one-hot vector *v*_*c*_, a vector of zeros except for the position corresponding to the ID of the concept. The ontology embedding layer transforms these sparse vectors into dense vectors, known as embeddings, through an embedding matrix $M \in \mathbb {R}^{D \times C}$, where *D* is the dimensionality of the embedding layer and *C* is the number of concepts of the ontology. Then, the output of the embedding layer is given by

$$f(c) = M \cdot v_{c} $$

In our experiments, we set the dimensionality of the ontology embedding layer as 50, and initialized its values randomly. Then, these values were tuned during training through back-propagation.

**Fig. 3:**
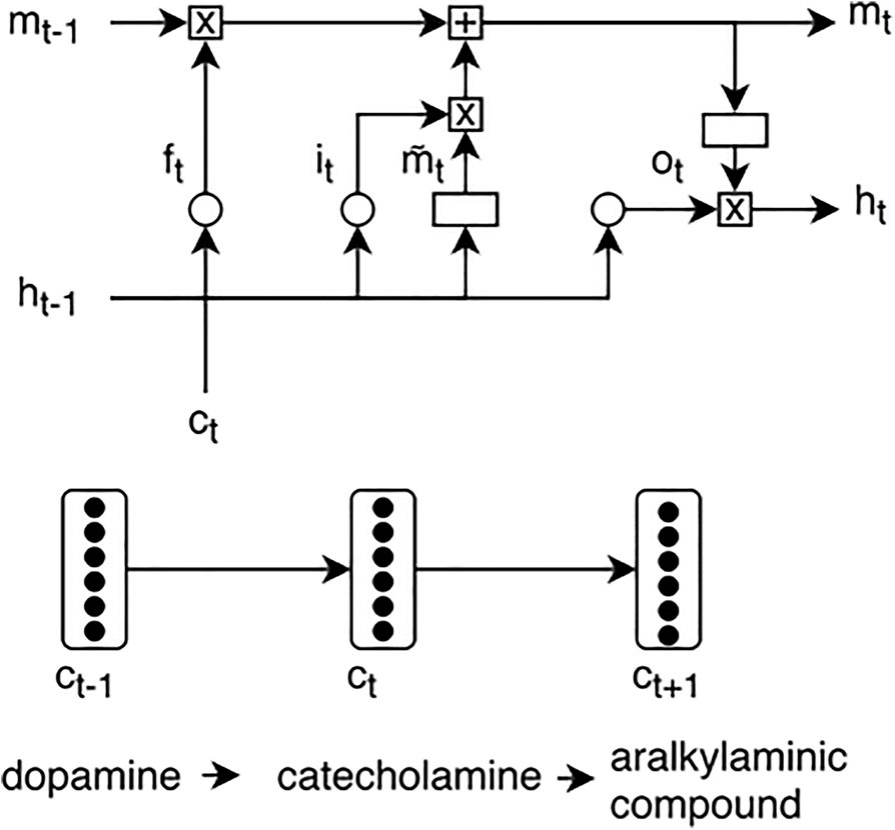
BO-LSTM unit, using a sequence of ChEBI ontology concepts as an example. Circle refers to sigmoid function and rectangle to tanh, while “x” and “+” refer to element-wise multiplication and addition. *h*: hidden unit; $\tilde {m}$: candidate memory cell; *m*: memory cell; *i* input gate; *f* forget gate; *o*: output gate

The sequence of vectors representing the ancestors of the terms is then fed into the LSTM layer. Figure 3 exemplifies how we adapted this architecture to our model, using a sequence of ontology concepts as input. After the LSTM layer, we use a max pool layer which is then fed into a dense layer with a sigmoid activation function. We experimented with bypassing this dense layer, obtaining inferior results. Finally, a softmax layer outputs the probability of each class.

Each configuration of our model was trained through mini-batch gradient descent with the Adam algorithm [46] and with cross-entropy as the loss function, with a learning rate of 0.001 We used the dropout strategy [47] to reduce overfitting on the trained embeddings and weights. We used a dropout rate of 0.5 on every layer except the penultimate and output layers. We tuned the hyperparameters common to all configurations using only the word embeddings channel on the validation set. Each model was trained until the validation loss stopped decreasing. The experiments were performed on an Intel Xeon CPU (X3470 @ 2.93 GHz) with 16 GB of RAM and on a GeForce GTX 1080 Ti GPU with 11GB of RAM.

The ChEBI and WordNet embedding layers were trained along with the other layers of the network. The DDI corpus contains 1757 of the 109k concepts of the ChEBI ontology. Since this is a relatively small vocabulary, we believe that this approach is robust enough to tune the weights. For the size of the WordNet embedding layer, we used 50 as suggested by Xu et al. [17], while for the ChEBI embedding layer, we tested 50, 100 and 150, obtaining the best performance with 50.

### Baseline models

As a baseline, we implemented a model based on the SDP-LSTM model of Xu et al. [17]. The SDP-LSTM model makes use of four types of information: word embeddings, part-of-speech tags, grammatical relations and WordNet hypernyms, which we refer to as channels. Each channel uses a specific type of input information to train an LSTM-based RNN layer, which is then connected to a max pooling layer, the output of the channel. The output of each channel is concatenated, and connected to a densely-connected hidden layer, with a sigmoid activation function, while a softmax layer outputs the probabilities of each class.

Xu et al. show that it is possible to obtain high performance on a relation extraction task using only the word representations channel. For this reason, we use a version of our model with only this channel as the baseline. We employ the previously mentioned pre-trained word embeddings as input to the LSTM layer.

Additionally, we make use of WordNet as an external source of information. The authors of the SDP-LSTM model showed that WordNet contributed to an improvement of the F1-score on a relation extraction task. We use the tool developed by Ciaramita and Altun [35] to obtain the WordNet classes of each word according to 41 semantic categories, such as “noun.group” and “verb.change”. The embeddings of this channel were set to be 50-dimensional and tuned during the training of the model.

We adopted a second baseline model to make a stronger comparison with other DDI extraction models, based on the model presented by Zhang et al. [48]. Their model uses the sentence and SDP of each instance to train a hierarchical LSTM network. This model is constituted by two levels of LSTMs which learn feature representations of the sentence and SDP based on word, part-of-speech and distance to entity. An embedding attention mechanism is used to weight the importance of each word to the two entities that constitute each pair. We kept the architecture and hyperparameters of their model, and added another type of input, based on the common ancestors and concatenation of each entity’s ancestors. We applied the same attention mechanism, so that the most relevant ancestors have a larger weight on the LSTM. We ran the original Zhang et al. model to replicate the results, and then ran again with ontology information.

## Results

We evaluated the performance of our BO-LSTM model on the SemEval 2013: Task 9 DDI extraction corpus [49]. This gold standard corpus consists of 792 texts from DrugBank [50], describing chemical compounds, and 233 abstracts from the Medline database [51]. DrugBank is a cheminformatics database containing detailed drug and drug target information, while Medline is a database of bibliographic information of scientific articles in Life and Health Sciences. Each document was annotated with pharmacological substances and sentence-level DDIs. We refer to each combination of entities mentioned in the same sentence as a candidate pair, which could either be positive if the text describes a DDI, or negative otherwise. In other words, a negative candidate is a candidate pair that is not described as interacting in the text. Each positive DDI was assigned one of four possible classes: mechanism, effect, advice, and int, when none of the others were applicable.

In the context of the competition, the corpus was separated into training and testing sets, containing both DrugBank and Medline documents. We maintained the test set partition and evaluated on it, as it is the standard procedure on this gold standard. After shuffling we used 80% of the training set to train the model and 20% as a validation set. This way, the validation set contained both DrugBank and Medline documents, and overfitting to a specific document type is avoided. It has been shown that the DDIs of the Medline documents are more difficult to detect and classify, with the best systems having almost a 30 point F1-score difference to the DrugBank documents [52].

We implemented the BO-LSTM model in Keras, a Python-based deep learning library, using the TensorFlow backend. The overall architecture of the BO-LSTM model is presented in Fig. 2. More details about each layer can be found in the “Methods” section. We focused on the effect of using different sources of information to train the model. As such, we tuned the hyperparameters to obtain reasonable results, using as reference the values provided by other authors that have applied LSTMs to this gold standard [18, 19]. We first trained the model using only the word embeddings of the SDP of each candidate pair (Fig. 2a). Then we tested the effect of adding the WordNet classes as a separate embedding and LSTM layer (Fig. 2b) Finally, we tested two variations of the ChEBI channel: first using the concatenation of the sequence of ancestors of each entity (Fig. 2c), and second using the sequence of common ancestors of both entities (Fig. 2d).

Table 1 shows the DDI detection results obtained with each configuration using the evaluation tool provided by the SemEval 2013: Task 9 organizers on the gold standard, while Table 2 shows the DDI classification results, using the same evaluation tool and gold standard. The difference between these two tasks is that while detection ignores the type of interactions, the classification task requires identifying the positive pairs and also their correct interaction type. We compare the performance on the whole gold standard, and on each document type (DrugBank and Medline). The first row of each table shows the results obtained using an LSTM network trained solely on the word embeddings of the SDP of each candidate pair. Then, we studied the impact of adding each information channel on the performance of the model, and the effect of using all information channels, as shown in Fig. 2.

**Table 1:** Evaluation scores obtained for the DDI detection task on the DDI corpus and on each type of document, comparing different configurations of the model

	DDI test			DrugBank			Medline		
Configuration	P	R	F	P	R	F	P	R	F
Word embeddings	0.7551	0.6865	0.7192	0.7620	0.7158	0.7382	0.6389	0.377	0.4742
+ WordNet	0.716	0.6936	0.7046	0.7267	0.7143	0.7204	0.5800	0.4754	0.5225
+ Common Ancestors	**0.7661**	0.6738	0.7170	**0.7723**	0.7003	0.7345	**0.6667**	0.3607	0.4681
+ Concat. Ancestors	0.7078	0.7489	0.7278	0.7166	0.7578	0.7366	0.6032	**0.623**	**0.6129**
+ WordNet + Ancestors	0.6572	**0.8184**	**0.7290**	0.6601	**0.8385**	**0.7387**	0.5574	0.5574	0.5574

Evaluation metrics used: Precision (P), Recall (R) and F1-score (F). Each row represents the addition of an information source to the initial configuration

Boldface indicates the configuration with highest score for each measure

**Table 2:** Evaluation scores obtained for the DDI classification task on the DDI corpus and on each type of document, comparing different configurations of the model

	DDI test			DrugBank			Medline		
Configuration	P	R	F	P	R	F	P	R	F
Word embeddings	0.5819	0.5291	0.5542	0.5868	0.5512	0.5685	0.5000	0.2951	0.3711
+ WordNet	0.5754	0.5574	0.5663	0.5845	0.5745	**0.5795**	0.4600	0.3770	0.4144
+ Common Anc.	**0.5968**	0.5248	0.5585	**0.6045**	0.5481	0.5749	**0.5152**	0.2787	0.3617
+ Concat. Anc.	0.5282	0.5589	0.5431	0.5286	0.5590	0.5434	0.4921	**0.5082**	**0.5000**
+ WordNet + Anc.	0.5182	**0.6454**	**0.5749**	0.5171	**0.6568**	0.5787	0.4590	0.4590	0.4590

Evaluation metrics used: Precision (P), Recall (R) and F1-score (F). Each row represents the addition of an information source to the initial configuration

Boldface indicates the configuration with highest score for each measure

For the detection task, using the concatenation of ancestors results in an improvement of the F1-score in the Medline dataset, contributing to an overall improvement of the F1-score in the full test set. The most notable improvement was in the recall of the Medline dataset, where the concatenation of ancestors increased this score by 0.246. The usage of ontology ancestors did not improve the F1-score of detection of DDIs in the DrugBank dataset. In every test set, it is possible to observe that the concatenation of ancestors results in a higher recall while considering only the common ancestors is more beneficial to precision. Combining both approaches with the WordNet channel results in a higher F1-score.

Regarding the classification task (Table 2), the F1-score was improved on each dataset by the usage of the ontology channel. Considering only the common ancestors led to an improvement of the F1-score in the DrugBank dataset and on the full corpus, while the concatenation improved the Medline F1-score, similarly to the detection results.

**Fig. 4:**
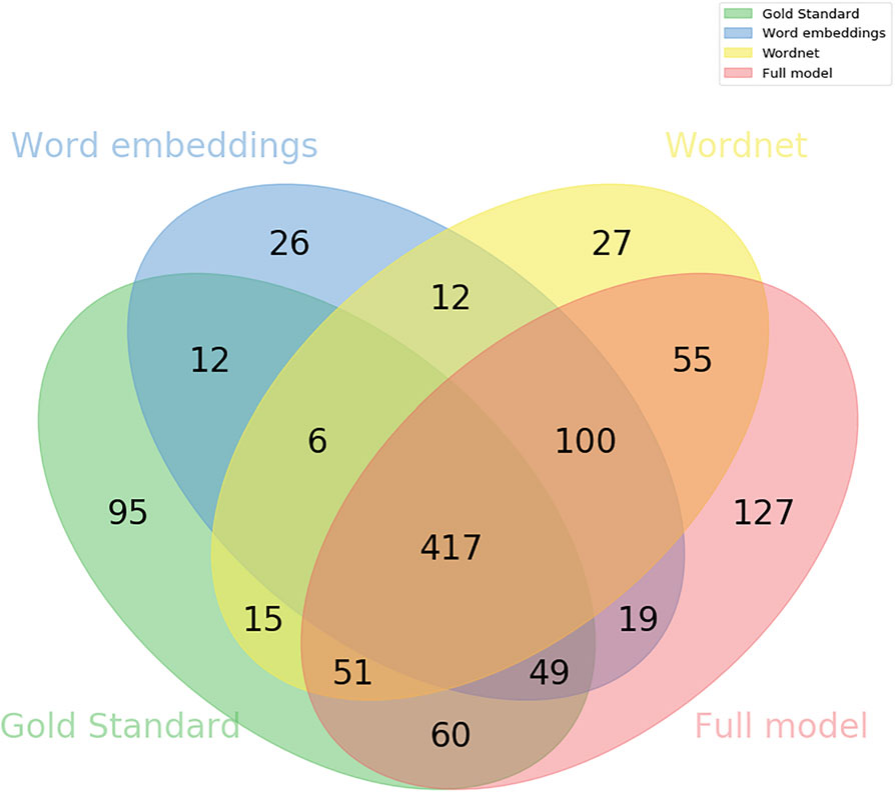
Venn diagram demonstrating the contribution of each configuration of the model to the results of the full test set. The intersection of each channel with the gold standard represents the number of true positives of that channel, while the remaining correspond to false negatives and false positives

**Fig. 5:**
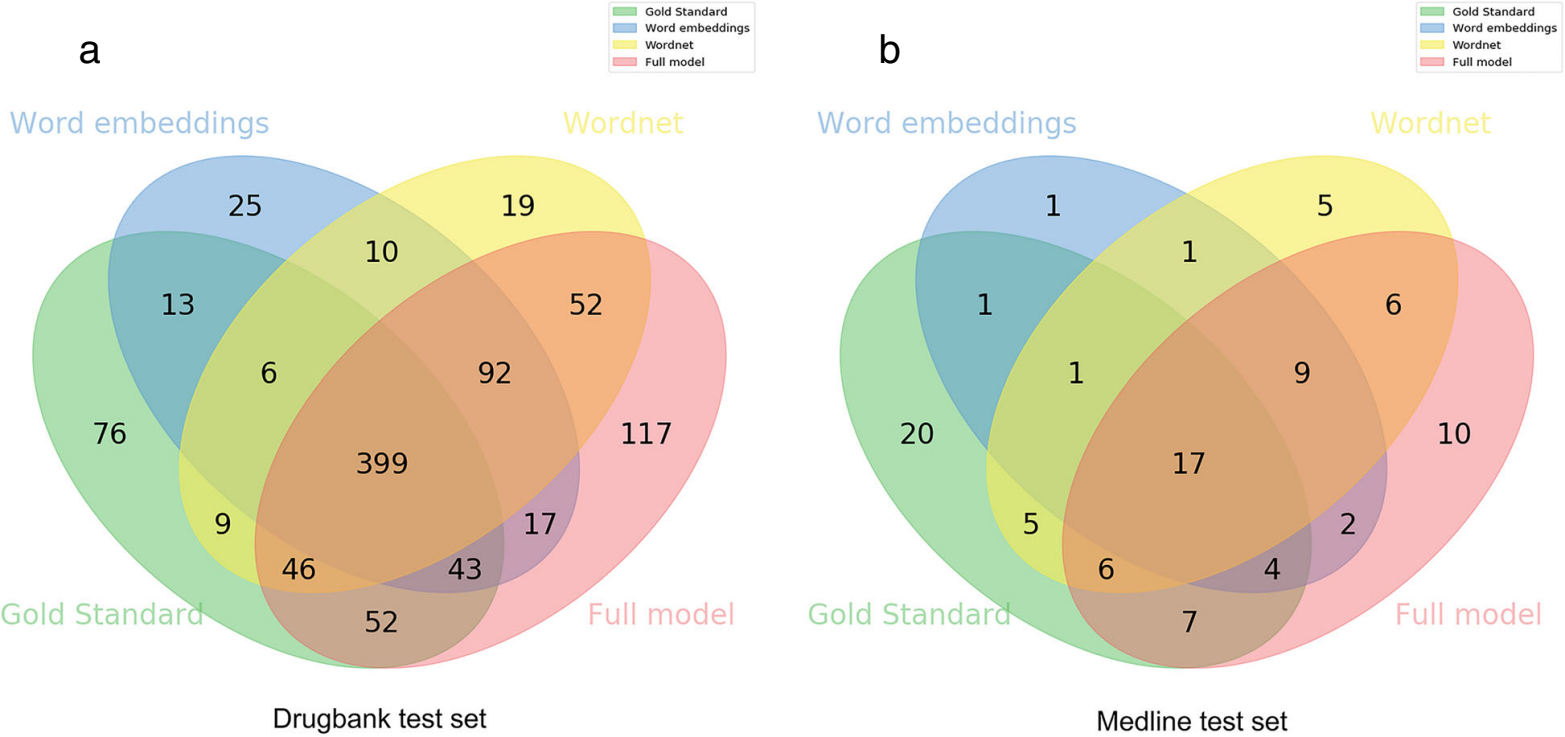
Venn diagram demonstrating the contribution of each configuration of the model to the DrugBank (**a**) and Medline (**b**) test set results. The intersection of each channel with the gold standard represents the number of true positives of that channel, while the remaining correspond to false negatives and false positives

To better understand the contribution of each channel, we studied the relations detected by each configuration by one or more channels, and which of those were also present in the gold standard. Figures 4 and 5 show the intersection of the results of each channel in the full, DrugBank, and Medline test sets. We compare only the results of the detection task, as it is simpler to analyze and show the differences in the results of different configurations. In Fig. 4, we can visualize false negatives as the number of relations unique to the gold standard and the false positives of each configuration as the number of relations that does not intersect with the gold standard. The difference between the values of this figure and the sum of their respective values in Fig. 5 is due to the system being executed once for each dataset. Overall 369 relations in the full test set were not detected by any configuration of our system, out of a total of 979 relations in the gold standard. We can observe that 60 relations were detected only when adding the ontology channels.

In the Medline test set, the ontology channel identified 7 relations that were not identified by any other configuration (Fig. 5b). One of these relations was the effect of quinpirole treatment on amphetamine sensitization. Quinpirole has 27 ancestors in the ChEBI ontology, while amphetamine has 17, and they share 10 of these ancestors, with the most informative being “organonitrogen compound”. While this information is not described in the original text, but only encoded in the ontology, it is relevant to understand if the two entities can participate in a relation. However, this comes at the cost of precision, since 10 incorrect DDIs were classified by this configuration.

To empirically compare our results with the state-of-the-art of the DDI extraction, we compiled the most relevant works on this task in Table 3. The first line refers to the system that obtained the best results on the original SemEval task [38, 53]. Since then, other authors have presented approaches for this task, most recently using deep learning algorithms. In Table 3 we compare the machine learning architecture used by each system, and the results reported by the authors. Since some authors focused only on the DDI classification task, we could not obtain the DDI detection results for those systems, hence the missing values. We were only able to replicate the results of Zhang et al. [48]. Since this system followed an architecture similar to ours, we adapted the model with our ontology-based channel, as described in the “Methods” section. This modification to the model resulted in an improvement of 0.022 to the F1-score. Our version of this model is also available on our page along with the BO-LSTM model.

**Table 3:** Comparison of DDI extraction systems

System	Architecture	Best F1-score
FBK-irst [38]	SVM	0.651
SCNN [18]	CNN	0.686
Joint AB-LSTM [19]	LSTM	0.6939
Att-BLSTM [22]	LSTM	0.773
DLSTM [20]	LSTM	0.6839
BR-LSTM [21]	LSTM	0.7115
Zhang et al. 2018 [48]	LSTM	0.729
Zhang et al. 2018 + BO-LSTM	LSTM	0.751

The architectures mentioned are Support Vector Machines (SVM), Convolutional Neural Networks (CNN) and LSTMs

We used the HP corpus to demonstrate the generalizability of our method. This case-study served only as a proof-of-concept, it was not our intent to measure the performance of the model, given the limited number of annotations and the dependence on the quality of using exact string matching to identify the genes. For example, we may have missed correct relations in the corpus, because they were not in the reference file or the gene name was not correctly identified.

Therefore, we used 60% (137 documents) of the corpus to train the model and 40% (91 documents) to manually evaluate the relations predicted with that model. For example, in the following sentence:


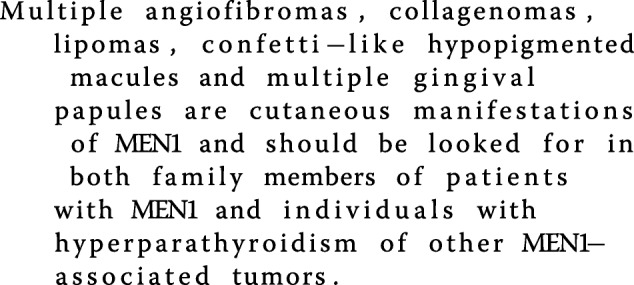
 the model identified the relation between the phenotype “angiofibromas” and the gene “MEN1”. One recurrently identified relation by our model that was not present on the phenotype-gene associations file is between the phenotype ’neurofibromatosis’ and the gene ’NF2’:


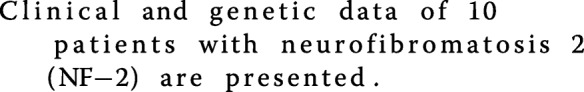


Despite this relation not being described in the previous sentence, it is predicted given its presence in the phenotype-gene associations files. With a larger number of annotations in the training corpus, we expect this error to disappear.

## Discussion

Comparing the results across the two types of documents, we can observe that our model was most beneficial to the Medline test set. This set contains only 1301 sentences from 142 documents for training, while the DrugBank set contains 5675 sentences from 572 documents. Naturally, the patterns of the DrugBank documents will be easier to learn than the ones of the Medline documents because more examples are shown to the model. Furthermore, the Medline set has 0.18 relations per sentence, while the DrugBank set has 0.67 relations per sentence. This means that DDIs are described much more sparsely than in the DrugBank set. This demonstrates that our model is able to obtain useful knowledge that is not described in the text.

One disadvantage of incorporating domain information in a machine learning approach is that it reduces its applicability to other domains. However, biomedical ontologies have become ubiquitous in biomedical research. One of the most successful cases of a biomedical ontology is the Gene Ontology, maintained by the Gene Ontology Consortium [54]. The Gene Ontology defines over 40,000 concepts used to describe the properties of genes. This project is constantly updated, with new concepts and relations being added every day. However, there are ontologies for more specific subjects, such as microRNAs [55], radiology terms [56] and rare diseases [57]. BioPortal is a repository of biomedical ontology, currently hosting 685 ontologies. Furthermore, while manually labeled corpora are created specifically to train and evaluate text mining applications, ontologies have diverse applications, i.e., they are not developed for this specific purpose.

We evaluate the proposed model on the DDI corpus because it is associated with a SemEval task, and for this reason, it has been the subject of many studies since its release. However, while applying our model to a single domain, we designed its architecture so it can fit any other domain-specific ontology. To demonstrate this, we developed a corpus of gene-phenotype relations annotated with Human Phenotype and Gene ontology concepts, and applied our model to it. Therefore, the methodology proposed can be easily followed to apply to any other biomedical ontology that describes the concepts of a particular domain. For example, the Disease Ontology [58], that describes relations between human diseases, could be used with the BO-LSTM model on a disease relation extraction task, as long as there is an annotated training corpus.

While we studied the potential of domain-specific ontologies based only on the ancestors of each entity, there are other ways to integrate semantic information from ontologies into neural networks. For example, one could consider only the ancestors with the highest information content, since those would be the most helpful to characterize an entity. The information content can be estimated either by the probability of a given term in the ontology or in an external dataset. Alternatively, a semantic similarity measure that accounts for non-transitive relations could be used to find similar concepts to the entities of the relation [59], or one that considers only the most relevant ancestors [60]. The quality of the ontology embeddings could also be improved by pre-training on a larger dataset, which would include a wider variety of concepts.

## Conclusions

This work demonstrates how domain-specific ontologies can improve deep learning models for classification of biomedical relations. We developed a model, BO-LSTM which combines biomedical ontologies with LSTM units to detect and classify relations in text. In this manuscript, we demonstrate that ontologies can improve the performance of deep learning techniques for biomedical relation extraction, in particular for situations with a limited number of annotations available, which was the case of the Medline dataset. Furthermore, we explored how it can be adapted to other relation extraction domains, for example, gene-phenotype relations. Considering that biomedical ontologies are openly available and regularly updated as the knowledge on the domain progresses, they should be considered important information sources for relation extraction.

## Abbreviations

ADR: Adverse Drug Reactions
ChEBI: Chemical Entities of Biological Interest
DDI: Drug-drug interactions
LSTM: Long short-term memory
NLP: Natural Language Processing
RNN: Recurrent Neural Networks
SDP: Shortest Dependency Paths
